# A prospective feasibility trial exploring novel biomarkers for neurotoxicity after isolated limb perfusion

**DOI:** 10.1177/02676591231213506

**Published:** 2023-11-07

**Authors:** Anna Corderfeldt Keiller, Markus Axelsson, Gudrun Bragadottir, Kaj Blennow, Henrik Zetterberg, Roger Olofsson Bagge

**Affiliations:** 1 Department of Cardiothoracic Surgery, 70712 Sahlgrenska University Hospital, Gothenburg, Sweden; 2 Department of Surgery, Institute of Clinical Sciences, 70712 Sahlgrenska Academy at the University of Gothenburg, Gothenburg, Sweden; 3 Wallenberg Centre for Molecular and Translational Medicine, 70712 University of Gothenburg, Gothenburg, Sweden; 4 Department of Clinical Neuroscience, Institute of Neuroscience and Physiology at Sahlgrenska Academy, 70712 University of Gothenburg, Gothenburg, Sweden; 5 Department of thoracic anesthesia and intensive care, 70712 Sahlgrenska University Hospital, Gothenburg, Sweden; 6 Department of Psychiatry and Neurochemistry, Institute of Neuroscience and Physiology, 70712 The Sahlgrenska Academy at the University of Gothenburg, Mölndal, Sweden; 7 Clinical Neurochemistry Laboratory, 70712 Sahlgrenska University Hospital, Mölndal, Sweden; 8 Department of Neurodegenerative Disease, UCL Institute of Neurology, Queen Square, London, UK; 9 UK Dementia Research Institute at UCL, London, UK; 10 Hong Kong Center for Neurodegenerative Diseases, Clear Water Bay, Hong Kong, China; 11 Wisconsin Alzheimer’s Disease Research Center, University of Wisconsin School of Medicine and Public Health, University of Wisconsin-Madison, Madison, WI, USA; 12 Department of Surgery, Sahlgrenska University Hospital, Gothenburg, Sweden

**Keywords:** extracorporeal circulation, isolated limb perfusion, neuroaxonal biomarkers, regional toxicity, simoa

## Abstract

**Background:**

Isolated limb perfusion (ILP) is a regional cancer treatment in which high-dose chemotherapy is administered in an isolated extremity. The main side effect is regional toxicity, which occasionally leads to nerve damage. Measuring neuroaxonal biomarkers, might be a method predicting such complications. Therefore, the primary aim of the study is to investigate if neuronal biomarkers are measurable and alters in an isolated extremity during ILP. Secondly, if postoperative regional toxicity, alterations in sensitivity, and/or muscle strength are correlated to the biomarker levels.

**Methods:**

Eighteen scheduled ILP-patients were included in the study. Glial fibrillary acidic protein (GFAP), neurofilament light (NfL), and tau concentrations were measured in plasma sampled preoperatively, at the start and end of the ILP, on days 3 and 30, using ultrasensitive Single molecule array (Simoa) technology. The patients were assessed by a physiotherapist pre- and postoperatively.

**Results:**

At ILP end, significantly higher NfL and tau levels were measured in the extremity than in the corresponding systemic circulation (NfL; 17 vs 6 ng/L, *p* < .01, tau; 1.8 vs 0.6 ng/L, *p* < .01), and the extremity levels were significantly increased at ILP end (NfL; 66 ± 37%, *p* < .001, tau; 75 ± 45%, *p* = .001). On days 3 and 30, significantly increased NfL and GFAP levels were measured systemically (NfL day 3: 69 ± 30%, *p* < .001; day 30: 76 ± 26%, *p* < .001; GFAP day 3: 33 ± 22%, *p* < .002; day 30: 33 ± 23%, *p* ≤ .004). Finally, no significant correlations were found between regional toxicity or between postoperative muscle or sensitivity decrease and biomarker release.

**Conclusion:**

During ILP, NfL and tau levels increased significantly. No obvious correlations were observed between biomarker release and regional toxicity or decreased muscle strength or sensitivity, although large-scale studies are warranted.

## Background

Isolated limb perfusion (ILP), pioneered in the 1950s by Creech and Krementz, is a regional treatment option for achieving high concentrations of a chemotherapy drug in an extremity affected by unresectable tumor/s, especially soft tissue sarcoma and melanoma, while avoiding systemic toxicity.^1,2^ For this purpose, the limb’s circulation is isolated from the systemic circulation and connected to an extracorporeal circulation (ECC) system, where a chemotherapeutic agent, usually melphalan or a combination of melphalan and TNF-alpha, is perfused through the limb under hyperthermia for 60–90 min.^3,4^ Currently, ILP is a safe and effective treatment choice for locally inoperable melanoma and sarcoma of the limb.^5,6^ This method has over time been refined and the current overall response rate (ORR) is >80%, with a complete response (CR) rate between 59% and 63%.^7,8^

Regional toxicity is a common side effect of ILP, affecting the treated limb, but is not often severe.^9^ ILP with melphalan results in erythema that appears between two and 5 days after the procedure and usually reaches its peak around 20 days later. It fades to a tan-like appearance after approximately 2–3 months.^10–14^ Regional toxicity is currently measured according to the Wieberdink classification.^15^ A meta-analysis by Sevilla-Ortega et al. in 2021, including a report on regional toxicity from 2546 ILP´s, showed a severe reaction with long-lasting side effects in only 2.7% of the patients (grade IV and V), but as many as 80% of the patients were assessed with mild to moderate reaction (grade II-III).^9^

Regional neurotoxicity is a well-known side effect of ILP, often resulting in different degrees of transient neuropathy.^12,16,17^ Symptoms such as motor and sensory impairment, burning sensations, tingling, and numbness often improve within weeks or months. However, severe irreversible paresis after ILP has also been reported.^16^ High doses of melphalan, higher temperatures, and longer perfusion times are correlated with higher neurotoxicity. High torniquet pressure for isolation of the extremities is also known to impair the nerves. Severe compression may stretch or cut the nerve fibers, leading to loss of excitability and conduction of the nerves distal to the lesion. This nerve fiber damage is a direct result of the applied pressure and not a secondary consequence of ischemia.^18–20^ Chemotherapy frequently induces sensory axonal polyneuropathy.^21^ However, in the retrospective study from Vrouenraets et al. including 367 patients, the degree of long-term neuropathy after ILP with melphalan was as low as 4%.^22^ Given that sensory symptoms are challenging to assess objectively in clinical practice, an easily accessible biomarker for chemotherapy-induced polyneuropathy (CIPN) holds the potential to improve early diagnosis.^21^

Neurofilaments are neuronal-specific intermediate filaments that determine axonal caliber, which in turn partly determines the conduction velocity along the axon.^23^ Neurofilaments are composed of neurofilament light protein in addition to the medium (NfM) and heavy protein counterparts (NfH). NfL is released into the cerebrospinal fluid (CSF) during axonal damage and has been shown to be elevated in numerous neurological diseases of the central nervous system (CNS) such as frontotemporal dementia, multiple sclerosis, and amyotrophic lateral sclerosis.^24–28^ NfL levels increases with a maximum between 7 and 10 days after neuroaxonal injury and declines slowly back to baseline between 200 and 400 days.^29^ Axons of the peripheral nervous system (PNS) also release NfL upon damage and have been shown in vasculitis and neuropathies such as Guillain Barré.^30,31^ Tau is a neuronal protein bound to microtubules that modulates neuronal and axonal stability.^32^ Increased levels of CFS tau are found in Alzheimer’s disease,^33^ and methods for measuring the phosphorylated form of tau protein (p-tau) also make it possible to differentiate Alzheimer’s disease from other neurodegenerative diseases.^34^ Novel ultrasensitive immunoassays allow for the measurement of total tau^35^ and p-tau.^36^ Increased tau levels in plasma have also been found in patients with inflammatory polyneuropathies.^31^ Tau increases within an hour after neuroaxonal injury and has a half-life in blood around 10 h^35^ Glial fibrillary acidic protein (GFAP) is the main intermediate filament protein in mature astrocytes and plays a crucial role in the astrocyte cytoskeleton, ensuring cell integrity and resilience.^37^ GFAP is involved in many important CNS processes, including cell motility and migration.^38,39^ GFAP is a traditional neuronal marker that is more strongly expressed in the elderly brain and is activated upon brain trauma or CNS degeneration.^39^ While NfL and tau are biomarkers for axonal injury (in central and peripheral nerves), GFAP is an astrocytic protein with specific expression in the central nervous system. GFAP peaks at 20 h after injury and slowly declines over 72 h.^40^

The method of ILP has been refined over time and is today a recommended, well tolerated, and efficient treatment.^5,14^ However, there are areas of improvement, such as regional toxicity, and the primary aim of this study is therefore to investigate if neuronal biomarkers are measurable and alters in an isolated extremity during ILP. Secondly, if postoperative regional toxicity, alterations in sensitivity, and/or muscle strength are correlated to the biomarker levels.

## Patients and methods

### Patients

This single-center feasibility trial included 18 patients scheduled for ILP between April 2019 and September 2020. The inclusion criteria were scheduled ILP, age ≥18 years, and written and informed consent. One patient had a preoperative stroke (<30 days prior to ILP) and was excluded from the study because this would affect the biomarker levels. The final analysis included 17 patients, of which the first ten patients also underwent assessment by a physiotherapist before ILP and 30 days after ILP. Ethical approval was obtained from the Swedish Ethical Board (Dr. 2019-01046) in March 2019, and conducted accordance with ICMJE Recommendations for the Protection of Research Participants. The trial was registered at ClinicalTrials.gov (NCT04460053).

### Clinical management

In all patients, anesthesia was induced with propofol (1.5 to 2.5 mg/kg), fentanyl (1.0 to 3 μg/kg), rocuronium (0.6 mg/kg) and maintained with sevoflurane. The ECC circuit was primed with 500 mL of ringer-acetate (Fresenius Kabi AB, Uppsala, Sweden), 100 mL of Tribonat (Fresenius Kabi AB), 100 mL Albumin 200 g/L (Baxalta, Illinois City, USA) and 2500 IU of heparin (LEO Pharma, Ballerup, Denmark) for leg perfusion. The priming solution for arm perfusion was the same as that for the leg except for one unit of packed red cells (approximately 250 mL) which was added together with 250 mL of ringer-acetate. The difference in prime regime between extremities was due to potential hemodilution anemia in the arms related to large prime volume/low surface area in the arms compared to the legs. The extremity was cannulated through the main vein and artery, isolated from the systemic circulation by an Esmarch tourniquet, and connected via tubing to a heart and lung machine (HLM). The chemotherapeutic drug (the alkylating agent melphalan, 13 mg/L limb volume in upper limb, and 10 mg/L limb volume in lower limb)^41^ was infused into the perfusion system for 20 min and then circulated in the extremity for an additional 40 min, with an ingoing blood temperature of 40°C. After a total perfusion time of 60 min, the chemotherapeutic drug was rinsed with 3000 mL of crystalloids. The ILP technique, HLM assembly, leakage monitoring, and temperature measurements were performed according to the clinical routine, as described previously in detail.^42^

### Blood sampling

Blood was sampled (5 mL) at five different timepoints during the procedure,

(1) The day before surgery (systemic circulation).
(2) Start of ILP.
  2(a) from extremity circulation (perfusate).
  2(b) from system circulation.
(3) End of ILP (70–100 min after start).
  3(a) from extremity circulation (perfusate).
  3(b) from system circulation.
(4) Day 3 postoperative (systemic circulation).
(5) Day 30 postoperative (systemic circulation).

Blood samples were collected into ethylenediaminetetraacetic acid (EDTA) tubes for plasma collection and centrifuged within 20–60 min. Plasma was separated, aliquoted, and stored at −80°C until biochemical analysis.

### Biomarker analysis

NfL, tau, and GFAP concentrations were measured in plasma using ultrasensitive Single molecule array (Simoa) technology and commercially available kits on an HD-X instrument (Quanterix, Billerica, MA, USA). All samples were analyzed in a single run at the Clinical Neurochemistry Laboratory at the University of Gothenburg, Mölndal, Sweden, by board-certified laboratory technicians blinded to clinical data using a single batch of reagents for each assay. The intra-assay coefficient of variation was <10% for all assays.

### Assessment by physiotherapist

The first 10 patients underwent a physiotherapist assessment preoperatively and at the return visit (day 30), where muscle strength (dorsal flexion of foot, knee flexion, knee extension for lower extremity and elbow flexion, and elbow extension for upper extremity) was measured using a N meter with a handheld dynamometer. Deep and superficial sensitivity in the foot and hand were assessed with blunt and sharp objects and manually measured on a scale of 0–2 (0 = no sensitivity, 1 = reduced sensitivity, 2 = full sensitivity).

### Statistical evaluation

Data are presented as mean ± standard deviation (SD), and paired samples t-test was used for normally distributed parameters. Median and percentiles 25–75 and Wilcoxon signed-rank test for non-parametric parameters. Variables were tested for normality using the Shapiro-Wilk test. The Kruskal-Wallis test was conducted to examine differences in biomarker release by regional toxicity reaction, and Pearson correlation was used to measure the strength of the linear relationship between muscle strength and sensitivity by biomarker release. A probability level (*p*-value) of less than 0.05 was considered statistically significant. Data were analyzed using SPSS version 28 (IBM, Armonk, NY, USA).

## Results

Seventeen patients were included in the final analysis: 12 males (71%) and five females (29%), with a median age of 72 years (range 29–89 years). Melanoma was the most common diagnosis (76%). Twelve patients underwent ILP of the leg and five patients underwent ILP of the arm (Table 1).

**Table 1.** Baseline patient characteristics for total observations, leg and arm.

Patient characteristics	Leg and arm (*n* = 17)	Leg (*n* = 12)	Arm (*n* = 5)
Gender, age			
Male *n*, (%)	12 (71%)	8 (67%)	4 (80%)
Age *years*, median (range)	72 (29–89)	72 (29–89)	74 (54–89)
Body constitution			
Height *cm*, mean (SD)	173 ± 10	174 ± 12	170 ± 6
Weight *kg*, mean (SD)	84 ± 19	85 ± 14	81 ± 28
Total Body Surface Area *m*^*2*^, mean (SD)	1.97 ± 0.2	2.00 ± 0.2	1.90 ± 0.3
Diagnosis			
Melanoma *n,* (%)	13 (76%)	11 (92%)	2 (40%)
Sarcoma *n,* (%)	3 (18%)	1 (8%)	2 (40%)
Merkel cell carcinoma, *n* (%)	1 (6%)	0 (0%)	1 (20%)
Preoperative blood work			
GFAP *ng/L,* median (percentiles)	160 (85–229)	145 (82–222)	197 (81–344)
NfL *ng/L,* median (percentiles)	19 (14–27)	18 (13–26)	24 (16–46)
Tau *ng/L,* median (percentiles)	1.9 (1.2–3.0)	1.8 (1.2–2.8)	1.9 (1.0–4.0)
Hemoglobin *g/L,* mean (SD)	125 ± 16	124 ± 18	126 ± 15
Creatinine *µmol/L,* mean (SD)	77 ± 13	75 ± 12	82 ± 15
Potassium *mmol/L,* mean (SD)	3.9 ± 0.4	3.9 ± 0.4	3.9 ± 0.4
Lactate *mmol/L,* mean (SD)	1.1 ± 0.4	1.1 ± 0.3	1.2 ± 0.6
Mean arterial pressure *mmHg,* mean (SD)	77 ± 15	77 ± 18	75 ± 9
Oxygen parameters after induction			
PaO_2_ *kPa,* mean (SD)	22 ± 16	20 ± 11	24 ± 27
SaO_2_ *%*, mean (SD)	98 ± 2	98 ± 1	97 ± 3
Co-morbidity			
No-comorbidity *n*, (%)	3 (18%)	2 (17%)	1 (20%)
Diabetes *n*, (%)	6 (35%)	4 (33%)	2 (40%)
Hypertension *n*, (%)	9 (53%)	5 (42%)	4 (80%)
Hyperlipidemia *n*, (%)	3 (18%)	3 (25%)	0 (0%)
Heart failure or heart surgery *n*, (%)	5 (29%)	4 (33%)	1 (20%)
Other co-morbidity,^a^ (%)	12 (71%)	9 (75%)	1 (20%)

Mean and standard deviation, median and percentiles or range, or numbers (%). Partial pressure of oxygen (PaO_2_), Oxygen saturation (SaO_2_).

^a^Other co-morbidity: Rheumatoid arthritis (*n* = 1), Parkinson´s disease (*n* = 1), bladder cancer (*n* = 1), prostate cancer (*n* = 1), breast cancer (*n* = 1), meningioma (*n* = 1), depression (*n* = 2), hip- and/or knee replacement (*n* = 3), asthma (*n* = 1).

### Biomarker level during isolated limb perfusion

**Figure 1.**
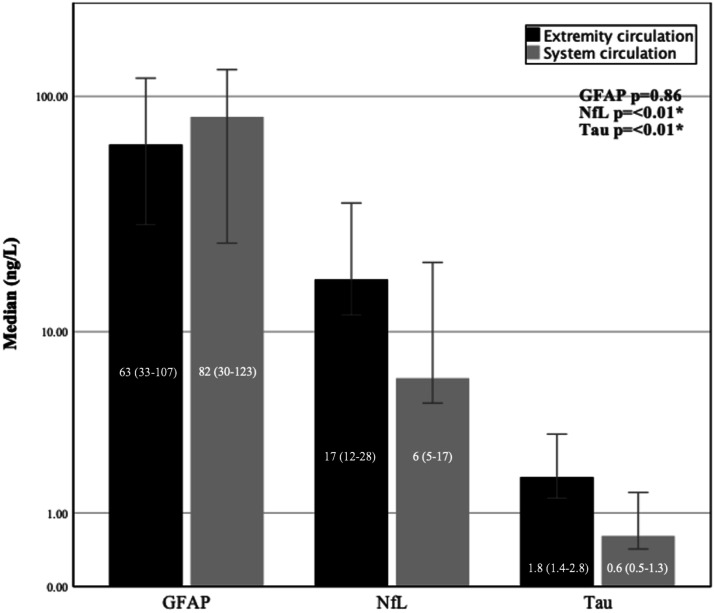
Clustered bar chart of median biomarkers in the extremity circulation compared to the systemic circulation at end of ILP. Logarithmic scale showing biomarkers in extremity circulation and systemic circulation at the end of ILP. System circulation is compensated for same hemodilution as perfusate due to priming volume in HLM. Wilcoxon signed ranks test. Error bars: CI 95%. *<0.05, **<0.005, ***<0.001.

At the end of the 60-min perfusion, before rinsing of the chemotherapy, there were significantly higher median levels of NfL and tau measured in the extremity circulation compared to the levels measured in the systemic circulation (NfL; 17 (12–28) versus 6 ng/L (5–17), *p* < .01), (Tau; 1.8 (1.4–2.8) versus 0.6 ng/L (0.5–1.3), *p* < .01). No significant difference was detected in GFAP (63 (33–107) versus 82 ng/L (30–123), *p* = .86) (Figure 1).

**Figure 2.**
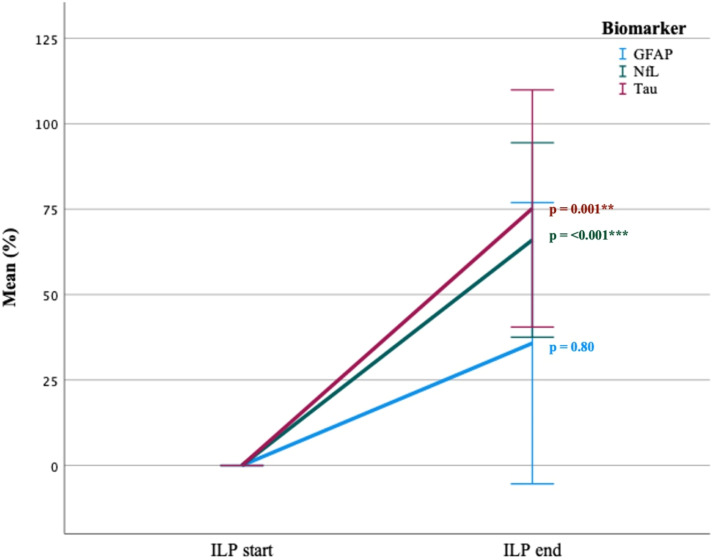
Multiple line of biomarkers mean change (%) from ILP start to ILP end in the isolated extremity. Error bars: 95% CI. Mean delta (%) of biomarkers between ILP start and ILP end. Paird samples T-test. *p* = *<0.05, **<0.005, ***<0.001.

A significant increase in mean levels of both NfL and tau was observed during perfusion (measured at ILP start and at ILP end) in the isolated extremity (NFL 0%–66% ± 37, *p* < .001 and tau 0%–75% ± 45, *p* = .001). GFAP levels did not change significantly (0%–36% ± 54, *p* = .80) (Figure 2).

### Biomarker levels after isolated limb perfusion

**Figure 3.**
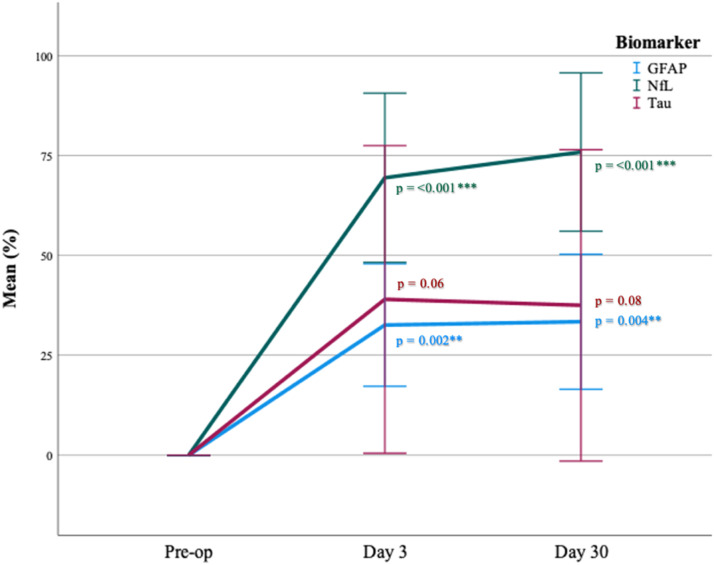
Multiple line of biomarkers mean change (%) in system circulation during postoperative follow up. Error bars: 95% CI. Mean change (%) of biomarkers in system circulation between “pre-op – day 3” and between “pre-op – day 30”. Paired samples T-test. *p* = *<0.05, **<0.005, ***<0.001. Compensated for the hemodilution occurring during perfusion.

During the postoperative follow-up, plasma sampled in the system circulation on days 3 and 30 showed significant mean elevation in both GFAP and NfL levels, with the highest level observed in NfL on day 30. Between “pre-op and day 3,” the GFAP level increased from 0%–33% ± 22, *p* < .002, and the NfL level increased from 0%–69% ± 30, *p* < .001. Between “pre-op and day 30,” the GFAP level increased from 0%–33% ± 23, *p* = .004 and the NfL level increased from 0%–76% ± 26, *p* < .001. No significant increase was observed in tau levels during follow up, neither on day 3 (0%–39% ± 55, *p* = .06) nor on day 30 (0 – 38 ± 52, *p* = .08) (Figure 3).

### Toxicity reaction and biomarker release

**Figure 4.**
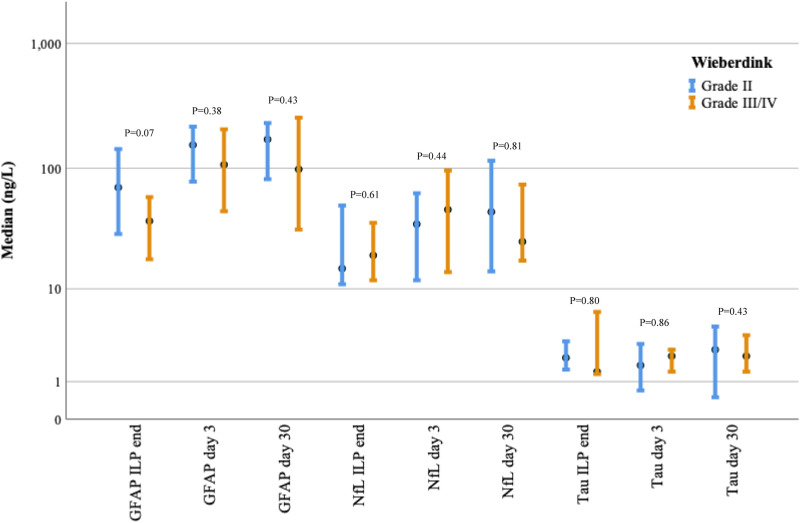
Clustered error bar of median biomarker release (ng/L) from “ILP end” → “Day 30” by Wieberdink regional toxicity reaction “day 30”. Logarithmic scale. Error bars: 95% CI. Kruskal-Wallis test for differences in mean rank between regional toxicity reaction and biomarker release, *p* = *<0.05, **<0.005, ***<0.001.

We hypothesized that an increase in the neuroaxonal biomarker level would lead to more severe regional toxicity after ILP according to the Wieberdink grading of limb toxicity,^15^ especially for patients with suspected temporary or permanent nerve injury (grade IV). At 30 days, there were 10 patients who had grade II, six patients had grade III and one patient had grade IV toxicity. When comparing grade II and grade III/IV, there was no significant difference between the level of biomarker release and toxicity reaction at any of the measured time points according to the Wieberdink grading scale (Figure 4).

### Correlation between muscle strength/sensitivity and biomarker release

Muscle strength and sensitivity were assessed by a physiotherapist the day before surgery and on the return visit day 30. A significant decrease in total muscle strength was seen at the return visit on day 30 (632 (424–779) versus 518 (366–603) Nm, *p* = .01), compared to before ILP. In addition, selective muscle strength, flexion, and extension were significantly decreased postoperatively compared with the preoperative levels (Table 2). There was no significant difference between the pre- and postoperative assessments of either deep or shallow sensitivity (Table 2).

**Table 2.** Physiotherapist assessment of muscle strength and sensitivity “preoperative” and “day 30 postoperative”.

Physiotherapy assessment	*n*	Preoperative	Day 30	*p*-value
Total muscle strength *Nm*	10	632 (424–779)	518 (366–603)	0.01*
Dorsal flexion foot *Nm*	8	192 (143–237)	154 (109–223)	0.04*
Knee flexion *Nm*	8	131 (101–165)	98 (76–116)	0.02*
Knee extension *Nm*	8	280 (184–372)	253 (124–333)	0.03*
Elbow flexion *Nm*	2	201 (107–195)	194 (119–172)	0.66
Elbow extension *Nm*	2	126 (45–145)	145 (82–136)	0.66
Total sensitivity *Points 0–4*	10	4.00 (3.75–4.00)	4.00 (3.00–4.00)	1.00
Deep sensitivity arm/leg *Points 0–2*	10	1.75 (2.00–2.00)	2.00 (2.00–2.00)	0.56
Shallow sensitivity arm/leg *Points 0–2*	10	2.00 (2.00–2.00)	2.00 (1.00–2.00)	0.16

Median and percentiles 25–75. Newton-meter (Nm). Wilcoxon signed ranks test. *p* = *<0.05, **<0.005, ***<0.001.

A significant correlation between GFAP release and increased sensitivity was observed postoperatively (R^2^ = 0.39, *p* = .03). No other significant correlation was observed between biomarker levels and changes in muscle strength and/or sensitivity between the pre- and postoperative assessments (Table 3).

**Table 3.** Correlation between biomarker release (ng/L) day 30 by delta muscle strength (%) and/or sensitivity (score) pre- and postoperative.

Physiotherapy assessment	*n*	GFAP *ng/L* R^2^/*p*-value	NfL *ng/L* R^2^/*p*-value	Tau *ng/L* R^2^/*p*-value
Pre- and postoperative delta % of total muscle strength	10	0.03/0.31	0.02/0.36	0.21/0.09
Pre- and postoperative delta % of total sensitivity	10	0.39/0.03*^a^	0.14/0.14	0.01/0.43

Pearson correlation and linear regression with R^2^. *p* = *<0.05, *<0.005, ***<0.001.

^a^Higher GFAP release correlates to increased postoperative sensitivity.

## Discussion

In this study, the overall aim was to investigate whether changes occurred in biomarker expression during isolated limb perfusion in an isolated extremity, and secondarily to analyze whether the level of biomarker release correlates with regional toxicity, changes in muscle strength, and/or sensitivity postoperatively. The main finding of this study was that NfL, and tau levels were significantly increased, more than twice, in the isolated extremity compared to the systemic circulation at the end of perfusion. During perfusion, the levels of NfL and tau increased by approximately 70% from start to end in the isolated extremity, while GFAP levels did not change significantly. In the systemic circulation during the follow-up on postoperative days 3 and 30, both GFAP and NfL levels also increased significantly, where NfL reached the measured biomarkers at the highest level from baseline on day 30 (76%). These findings indicate that NfL and tau are released from the peripheral nerves during ILP treatment. The absence of a change in GFAP concentration in the isolated extremity speaks against central nervous system effects during perfusion. However, the significant postoperative increase in GFAP expression during follow-up indicated that the central nervous system was affected by treatment. To the best of our knowledge, this is the first study to show that an isolated peripheral nerve exposed to chemotherapy during an ILP shows signs of neural damage.

Although we found a significant increase in the release of NfL and tau at the end of the perfusion in the extremity compared to the systemic circulation, we could not find a significant correlation between the level of biomarker release and regional toxicity using the Wieberdink regional toxicity grading. However, the sample size needed to establish such a correlation needs to be larger and will have to be an aim for future studies.

The physiotherapist’s assessment of the patients’ pre- and postoperative muscle strength and sensitivity showed a significant decrease in total muscle strength (consisting of flexion and extension of the foot, knee, and elbow) postoperatively compared to preoperative values; however, no difference was seen in sensitivity between the pre- and postoperative assessments. Although muscle strength significantly decreased postoperatively, this could not be explained by a significant correlation between changes in muscle strength and biomarker release. Furthermore, there was no significant correlation between sensitivity changes and biomarker release observed in our study. Other studies have suggested that NfL levels are a valuable biomarker of neuroaxonal injury in chemotherapy-induced peripheral neuropathy, which occurs mainly when paclitaxel is used to treat breast and ovarian cancer.^21,43,44^ We were unable to demonstrate the same correlation in our study. Perhaps a larger study population or usage of electroneuronography in assessing the nerve pre- and postoperatively could have detected finer alterations.

## Conclusion

At the end of the ILP, we found significantly higher levels of both tau and NfL in the extremities than in the circulatory system. We also found that the levels of NfL and tau increased significantly from the start to the end of the ILP in isolated extremities. No correlation was found between the biomarkers and regional toxicity or muscle and/or sensitivity assessment. An isolated peripheral nerve exposed to chemotherapy during an ILP shows signs of neuronal damage. Further research with a larger study population is warranted.

## Data Availability

Requests for the data is subject to the General Data Protection Regulation, the Swedish Act SFS 2018:2018, the Swedish Data Protection Act, the Swedish Ethical Review Act, and the Swedish Public Access to Information and Secrecy Act. Requests must be reviewed by the University of Gothenburg. Further information is available in the dataset publication.^45^
